# Familiar and unfamiliar face recognition in crested macaques (*Macaca nigra*)

**DOI:** 10.1098/rsos.150109

**Published:** 2015-05-27

**Authors:** Jérôme Micheletta, Jamie Whitehouse, Lisa A. Parr, Paul Marshman, Antje Engelhardt, Bridget M. Waller

**Affiliations:** 1 Centre for Comparative and Evolutionary Psychology, Department of Psychology, University of Portsmouth, Portsmouth, UK; 2 Center for Translational Social Neuroscience, Silvio O. Conte Center for Oxytocin and Social Cognition, Department of Psychiatry and Behavioral Sciences, Emory University, Atlanta, GA, USA; 3 Yerkes National Primate Research Center, Emory University, Atlanta, GA, USA; 4 Junior Research Group for Primate Sexual Selection, German Primate Center, Göttingen, Germany; 5 Courant Research Centre for the Evolution of Social Behaviour, Georg-August University, Göttingen, Germany

**Keywords:** crested macaques, dominance, familiarity, individual recognition, matching-to-sample, social bond

## Abstract

Many species use facial features to identify conspecifics, which is necessary to navigate a complex social environment. The fundamental mechanisms underlying face processing are starting to be well understood in a variety of primate species. However, most studies focus on a limited subset of species tested with unfamiliar faces. As well as limiting our understanding of how widely distributed across species these skills are, this also limits our understanding of how primates process faces of individuals they know, and whether social factors (e.g. dominance and social bonds) influence how readily they recognize others. In this study, socially housed crested macaques voluntarily participated in a series of computerized matching-to-sample tasks investigating their ability to discriminate (i) unfamiliar individuals and (ii) members of their own social group. The macaques performed above chance on all tasks. Familiar faces were not easier to discriminate than unfamiliar faces. However, the subjects were better at discriminating higher ranking familiar individuals, but not unfamiliar ones. This suggests that our subjects applied their knowledge of their dominance hierarchies to the pictorial representation of their group mates. Faces of high-ranking individuals garner more social attention, and therefore might be more deeply encoded than other individuals. Our results extend the study of face recognition to a novel species, and consequently provide valuable data for future comparative studies.

## 2. Introduction

Faces and the information they convey are of crucial importance for primates. Most, if not all primates, including humans, rely on faces to identify individuals and extract socially relevant information such as gender, age and emotional state [1–4]. In addition, primates often live in complex social groups with highly individualized and complex social relationships, and so need to accurately distinguish between individuals. Several species can use visual cues present on the face to discriminate individuals from still photographs [5]. Manipulation of the stimuli allows us to get insight into the cognitive mechanisms underlying this expertise and the extent to which it is shared across species [6,7]. However, with few notable exceptions (e.g. [8–10]), most studies are restricted to a limited subset of species (mostly chimpanzees, *Pan troglodytes*, and rhesus macaques, *Macaca mulatta*) tested with faces of unfamiliar individuals, and often housed alone or in pairs. Regardless of what causes these biases in the existing literature (e.g. limited access to other species and/or to group-housed animals), they undoubtedly hinder the scope of a comparative approach to the evolution of face processing and its function [5].

One well-known and interesting phenomenon in human face processing is that humans do not perform equally well when tested with familiar and unfamiliar faces. While we are usually extremely good at recognizing familiar faces, accuracy is generally lower and reaction-time higher with unfamiliar faces [11]. This discrepancy is especially apparent when subjects have to match non-identical images: changes in orientation, viewpoint or lighting strongly affect our ability to recognize unfamiliar but not familiar individuals [11,12]. These differences have led scientists to propose a qualitative difference between familiar and unfamiliar faces: unfamiliar faces seem to be processed using low-level image description (i.e. they are coded as pictures) while visual representation of familiar faces is accompanied by rich, semantic, episodic and emotional information about the person represented on the picture, which we need for effective social interactions [11,13–15]. Given that non-human primates also live in complex and fluid societies, and given the extent of their social knowledge (e.g. [16,17]), it would seem logical that they too could have the same advantage as humans with familiar faces. Very few studies have addressed this question and the results remain equivocal.

Chimpanzees tested with familiarized faces (i.e. unfamiliar faces repeatedly shown to the subjects until they are familiar) exhibited better performances with familiarized faces than non-familiar faces when presented across different viewpoints [7], a pattern similar to the one observed in humans [12]. Capuchin monkeys (*Cebus apella*) could individuate familiar and unfamiliar individuals in an oddity task; however, they were more accurate with the unfamiliar ‘out-group’ faces than with familiar ‘in-group’ ones. In addition, one subject responded more quickly when discriminating out-group faces [9]. This could mean that capuchins did not benefit from the personal knowledge associated with familiar faces of group mates, which, therefore, raises the question of whether or not they processed the pictures of familiar faces as representations of the real individuals in the way that chimpanzees and humans do. In a follow-up study with the same individuals, subjects correctly identified one in-group member as odd among three out-group members, and vice versa [18]. The authors argued that the capuchins applied their personal knowledge of group membership to solve the task and therefore suggest that the subject in these experiments could use the pictures as representations of the real individuals (i.e. equivalence, *sensu* [19]). Similar conclusions are supported by cross-modal studies where subjects successfully matched the voice and face of familiar individuals [20–22], and a number of playback studies showed that some species seem to have mental representations of kin and dominance relationships within their group [16,23].

Another way to further explore the possibility that non-human primates can indeed use pictures as representations of real individuals and use their social knowledge in individual recognition tasks is to explore the pattern of answers made in matching-to-sample (MTS) experiments. Using group-housed individuals allows researchers to explore the importance of social variables such as the dominance hierarchy, kinship networks and variations in the strength of social bonds between individuals with a reasonable degree of ecological validity [24–26]. Such an approach can help us to gain a better understanding of the social influences on individual recognition and category formations [27,28]. If subjects perceive the photographs as representations of their group mates and conceive their relationships within the group, it is possible that the nature and quality of these social relationships affects the way the stimuli are encoded, thus affecting memory retrieval. If this is the case, we would expect to observe biases in subject performances, such that subjects might favour higher ranking individuals [29–31] and/or individuals with whom they share a strong social bond [25,32].

In this study, we tested crested macaques (*Macaca nigra*) in a series of MTS experiments building on previous studies conducted with rhesus macaques [33–35]. Like other macaque species, crested macaques live in multi-male multi-female groups reaching up to 100 individuals organized in matrilines and presenting a linear transitive dominance hierarchy [36,37]. However, crested macaques differ from the more commonly studied rhesus macaques in patterns of aggression, affiliation, dominance and nepotism, and consequently are considered more socially tolerant than rhesus macaques [36]. Despite being phylogenetically closely related, there are reasons to expect differences in the cognitive abilities of rhesus and crested macaques. Recent studies highlighted the potential influence of species social style on cognition. For example, in a cooperative task, social tolerance allows bonobos (*Pan paniscus*) to outperform chimpanzees [38]. In gaze following tasks, more tolerant species of macaques seemed highly sensitive to gaze cues given by close affiliates [25,39] while more despotic species' performances were affected by social status [30,40]. Finally, the cognitive abilities of crested macaques remain virtually unknown and so this study will provide a valuable insight into the cognitive abilities of a socially tolerant and understudied macaque species.

In a first task, the crested macaques matched different photographs of the same unfamiliar individuals. In a second task, the same individuals matched different photographs of familiar individuals with whom they interact on a daily basis. The order of the tasks was counterbalanced across subjects. We compared individual performances (accuracy and reaction time) when matching unfamiliar and familiar faces. Previous studies found that rhesus macaques prefer to look at faces of high-ranking individuals [29,31] and selectively follow the gaze of high social status conspecifics [30], while crested macaques favour close affiliates [25]. Therefore, we also examined potential social biases (i.e. relative dominance status between the individuals depicted in a trial, and strength of the social bond between the subject and individuals depicted in a trial) conflicting with the MTS rule.

## 3. Material and methods

### 3.1 Subjects

Data were collected from three adult crested macaques (one male, *Bai*, 9 years old; and two females, *Sat*, 7 years old, and *Dru*, 12 years old) belonging to a social group of five individuals housed at Marwell Zoo, Winchester, UK (http://www.marwell.org.uk). The macaques had access to a three-part enclosure: one indoor enclosure (5×5×5 m), one outdoor enclosure (10×5×5 m) and one outdoor island (approx. 15×15 m). All parts of the enclosure were equipped with vertical and horizontal wooden structures, ropes, trees and various enrichment devices. The macaques were fed daily with commercial monkey pellets, fruits, vegetables, seeds and nuts before and after the experiments. Water was freely available.

The dominance hierarchy of the group was established through the creation and analysis of a matrix of dyadic interactions based on the outcome of decided aggressive interactions such as displacements, supplantations and unidirectional aggression [25,41]. Similar measures of dominance were obtained from previous studies for the unfamiliar individuals used as stimuli [32,42]. We calculated a composite social index (CSI) to characterize the strength of the social bond between the individuals [43]. The CSI was calculated using the frequency of grooming interactions and the frequency of sitting in proximity within a dyad, and reflects the strength of the social bond between two individuals [25,43].

### 3.2 Apparatus

**Figure 1.**
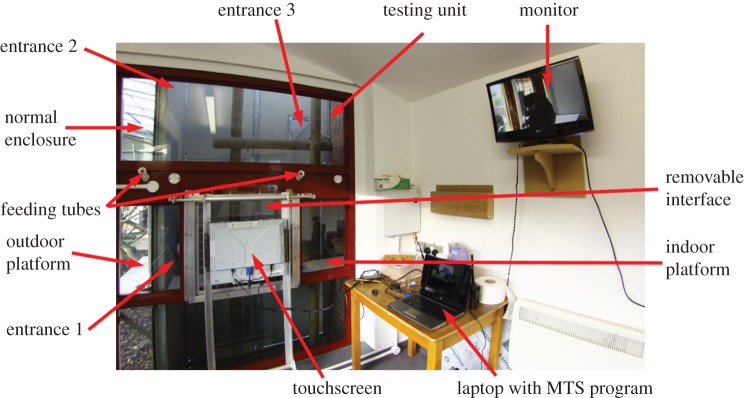
Overview of the Macaque Study Centre. The macaques sit on the outdoor platform until they are granted access to the testing unit. Then they sit on the indoor platform and can use the touchscreen through the interface.

The macaques had unrestricted access to a testing unit (approx. 1.5×2×5 m) adjacent to the outdoor enclosure and equipped with a computerized touchscreen (Elo 1939L 19-inch Open-Frame Touchmonitor, Elo Touch Solutions Limited, Swindon, UK), a platform for the macaques to sit on, several poles to facilitate the entrance and exit from the unit, and feeding tubes to dispense food rewards (figure 1). A video camera was placed behind the animals and a direct video stream was displayed on a monitor in the experimenter area. The touchscreen was linked to a laptop computer where the experimenter could run custom-made programs (programmed in Visual Basic with Microsoft Visual Studio 2010). The program recorded the identity of the individual taking part in the test, the type of the stimuli used in the task, the latency to complete a trial (time from starting a trial to selecting a response, hereafter reaction time) and whether the response was correct or not. The animals could enter and exit the unit at any time but could only access the touchscreen when an experimenter was present. Tests were carried out 3 days per week. For dietary reasons, the number of trials was limited to 48 per individual and per day. Because the macaques could exit the unit at any time, they often started a session without finishing it. In these cases, the session was saved and resumed to the last unfinished trial whenever the individual came back in the testing unit.

### 3.3 Matching-to-sample procedure

**Figure 2.**
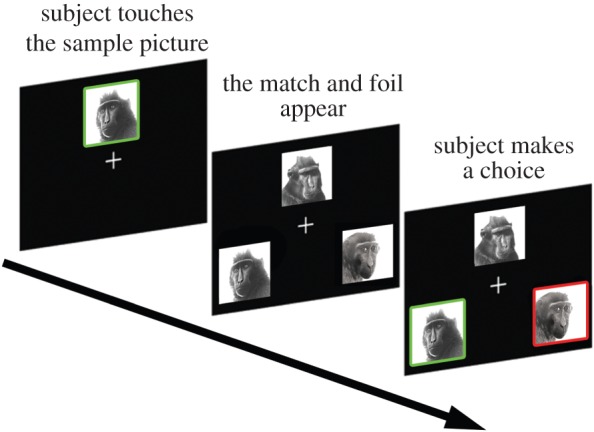
Trial progression of a MTS trial. The macaques were required to first touch the sample picture three times to ensure they are oriented towards the sample picture. Then the match and foil pictures appear in a triangular configuration. If the macaques choose the matching picture, they are given a food reward and have to wait 2 s until the next trial. If they choose the foil, they obtain no reward and have to wait 8 s until the next trial.

We employed the MTS format [34,44] for all the tasks presented here (figure 2). Details of the training procedure are given in the electronic supplementary material. First, we required subjects to orient towards a single image on the screen (hereafter, the sample) by touching it three times in rapid succession. The sample appeared randomly in a central position on the top, bottom, left or right side of the screen. After the initial response, two comparison images were displayed on the screen, on the opposite side of the sample, forming a triangular configuration. The sample remained visible after the appearance of the comparison images (simultaneous MTS). Then, the subject had to choose the comparison image that matched the sample (hereafter, the match). If the response was correct, it was followed by an inter-trial interval of 2 s during which the subject received a food reward (peas, sweet-corn or cereal). If the subject chose the incorrect comparison image (hereafter, the foil), it did not receive any food reward and the inter-trial interval lasted 8 s.

Following Parr *et al*. [34], we trained the macaques to perform the computerized MTS task using geometric shapes, simple clip-arts and identical pictures of unknown conspecifics (we used pictures of crested macaques held in various UK zoo collections during training and pictures of wild individuals during the tests). Although all individuals could access the testing unit and participate, the three highest ranking individuals tended to monopolize the touchscreen and consequently only these three individuals participated in this initial training stage and in the subsequent experiments. For ethical reasons, we did not isolate the other subjects for testing and therefore did not force them to participate in the study.

### 3.4 Stimuli

For the unfamiliar individual recognition (UIR) trials, the stimuli were high-quality photographs of unfamiliar crested macaques' neutral faces taken in the Tangkoko Nature Reserve, North Sulawesi, Indonesia (Macaca Nigra Project field site: http://www.macaca-nigra.org) with a digital SLR camera. For the familiar individual recognition (FIR) trials, the same camera was used to take pictures of five individuals, including our three subjects and the two other group members. We cropped each photograph around the face, converted it to grey scale and resized it to a height and width of 300 pixels (electronic supplementary material, figure S1). We standardized the background across photographs using the *brush tool* in Adobe Photoshop CS5 while keeping as much detail of the individual's crest as possible, as it could be an important, individually specific physical feature (electronic supplementary material, figure S1). Within each stimulus set (sample, match and foil), we matched the brightness and contrast using the *colour match tool* of the same software (the statistics of one photograph are saved and used as a template for the other photographs).

### 3.5 Procedure

#### 3.5.1 Unfamiliar individual recognition

In an UIR trial, the sample and the match were two different photographs of the same unfamiliar conspecific, while the foil depicted another unfamiliar conspecific. These images are collectively referred to as a stimulus set and represent one trial. We used 12 unique stimulus sets (36 unique pictures of 24 different individuals) in this task. Six stimulus sets featured adult females while the other six featured adult males. We controlled for the sex and approximate age of the individuals (the sample, match and foil always represented individuals of the same sex and age category). The trials contained an equal mix of similar and dissimilar head orientation between the sample and the foil. In control trials, the sample and match were identical photographs of an unfamiliar conspecific (different to the ones used in the test trials) and the foil depicted an unfamiliar rhesus macaque. These control trials were included to ensure that the subject was still conforming to the MTS rule and would always receive some food rewards to encourage participation in the task. All three subjects were highly proficient with this type of trial.

**Figure 3.**
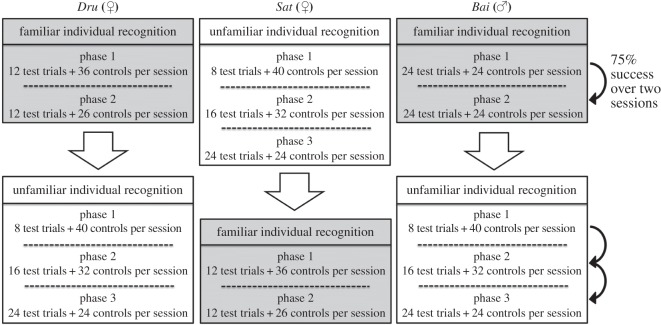
Diagram of the experimental design. To avoid order effect, one individual (*Sat*) started with the UIR task, the other two (*Bai* and *Dru*) with the FIR task. As the number of available familiar individuals was limited, the number of trials differs between the UIR and FIR tasks. In the FIR task, the number of trials also differs according to the sex of the subject (see Procedure section for details).

Each subject was tested three days a week and could complete one session of 48 trials per day (24 unique trials including both test and control trials, repeated twice). Following the procedure described in Parr *et al.* [34], the UIR trials were presented in three phases (figure 3). In these phases, trials were added cumulatively so that trials 1 to 4 were presented first. When the subjects reached a mean of 75% of success on this block of trials, then trials 5 to 8 were added and finally trials 9 to 12. The number of control trials was reduced each time new UIR trials were included to maintain the maximum of 48 trials per session. Using this procedure, we could check whether subjects' performances increased after repeated exposure to the same trials, which could indicate a learning effect. Binomial *z*-scores are often used to determine the chance level in these experiments (e.g. [33,34]), but with so few trials this would lead to very stringent criteria (87% correct for four UIR trials repeated twice). So instead we chose the criterion of 75%, which is still fairly stringent (compared with the 50% threshold often used) and also facilitates comparison with the performance of rhesus macaques [34]. Such high percentages of success were difficult to obtain for rhesus macaques tested in the same task [34].

#### 3.5.2 Familiar individual recognition

In a FIR trial, the sample and the match were two different photographs of the same familiar conspecific (i.e. member of the same social group as the subject), while the foil depicted another familiar conspecific. Each member of the group was presented as the sample and tested against all other group members. Because our group contained only one male, we only tested subjects with pictures of females. The male subject was tested with pictures of all four females (four possible samples with three possible foils=12 possible sets). The females were tested with pictures of all females not including themselves (three possible samples with two possible foils=6 possible sets). Within a session, each familiar individual was presented three times but on different pictures. We used 12 unique stimulus sets (36 unique pictures of four different individuals) in this task. The FIR trials contained an equal mix of similar and dissimilar head orientations between the sample and the foil. In control trials, the sample and match were identical photographs of an unfamiliar conspecific (other than the ones used in the test trials) and the foil depicted an unfamiliar rhesus macaque.

The subjects were tested three times a week and could complete one session of 48 trials per day (24 unique trials including test and control, repeated twice). The macaques had free access to the testing unit and could leave when they wanted to. In a first phase, we presented the subjects with 24 unique FIR trials for the male, six for the females. Once they exceeded 75% of success, they were moved to a second phase similar to the first one but with an entirely novel set of pictures and again tested until they reached the 75% criterion (figure 3).

### 3.6 Data analysis

To examine the relationship between the explanatory variables and the occurrence of success (binary response: correct/incorrect), we used generalized linear mixed models (GLMMs) with a binomial error structure and logit link function. In a first model, we investigated (i) whether the performances of the subjects differed between the UIR and FIR task, (ii) for each task, whether their performances differed for the different set of stimuli we used and (iii) whether their performances improved after repetition of the same trials. The type of task (categorical: UIR versus FIR), a stimulus set identifier (categorical: 1 to 24) and the number of repetitions for a given stimulus set (continuous) were included as predictors. To take into account repeated measures for the same individuals, subjects' identity was entered as a random factor [45]. A similar model with a Gaussian error structure was built to examine the variation of subjects' reaction time.

We then further investigated subjects' responses in the FIR task. This model (binomial error structure and logit link function) was built to investigate possible biases in accuracy depending on (i) the social status of the sample individual compared with that of the foil individual and (ii) the social bond existing between the subject and the sample individual. The response for each trial (correct/incorrect) was set as the dependent variable. The social status of the individual presented as the sample relative to that of the foil individual (dominant/subordinate) and whether the sample individual was strongly affiliated with the subject (affiliate/non-affiliate) were entered as categorical predictors. Again, identity of the subjects was used as a random factor. A similar model with a Gaussian error structure was built to examine the effects of the social variables on subjects' reaction time.

We fitted GLMMs using the functions *glmer* and *lmer* provided by the package *lme4* [46] for R v. 2.15.1 [47]. The *p*-values from GLMMs with Gaussian error structure were calculated based on Markov chain Monte Carlo (MCMC) sampling and derived using the function *pvals.fnc* of the package *language* [48]. To assess the overall significance of a model, we compared it to a null model including only the intercept and the random variables by performing a likelihood ratio test (LRT, function *anova*) comparing the log-likelihoods of both models [49,50]. We considered significant effects only if the model with predictors was more informative than the null model. For categorical predictors with more than two levels (e.g. stimulus set), we assessed the overall significance of the predictor with a LRT comparing the full model to a model without that specific predictor. We report estimates and their standard error, alongside *p*-values. Estimates for categorical predictors represent the change in the dependent variable relative to the reference level, and so show the strength and direction of each level's influence on the dependent variable. Estimates for continuous variables represent the change in the dependent variable per unit of the predictor variable.

Because the macaques could come and go as they pleased, and because we did not include any time restriction in our program, some reaction times were extremely high. Visual inspection of the data revealed several potential outliers. Following Baayen & Milin [51], we removed five data points (out of 531) with absolute standardized residuals exceeding 2.5 s.d. Reaction times were log-transformed to approximate normal distribution. Visual inspection of the residuals revealed no obvious deviation from homoscedasticity or normality.

## 4. Results

### 4.1 Unfamiliar individual recognition

All three individuals participated in this experiment. Their performances are summarized in table 1. All individuals reached 75% success in the first phase after an average of 3.67 sessions (*Bai*: 3, *Dru*: 5, and *Sat*: 3). The macaques were then moved to the second phase. *Bai* reached 68.75% on session 4; *Dru* reached 62.50% on her first session. Both individuals failed to improve afterwards (probably due to poor motivation to participate in the task). Only *Sat* reached 75% success after two sessions. She was moved to the third and last phase and reached the 75% criterion on session 5. Not all individuals reached the 75% criterion, so we could not compare the number of sessions required to achieve this performance. However, there were no obvious consistent differences in the number of sessions each individual required to reach their maximum performances (table 1).

**Table 1.** Number of sessions and trials completed by each subject and summary of their performances in the unfamiliar (UIR) and familiar face recognition (FIR) tasks.

ID	task	no. stimulus sets	no. sessions	no. trials^*a*^	max. success^*b*^
*Bai*	UIR	4	3	24 (144)	75.00
*Dru*			5	40 (240)	75.00
*Sat*			3	24 (144)	87.50
*Bai*		8	7	112 (336)	68.75
*Dru*			5	80 (240)	62.50
*Sat*			2	32 (96)	75.00
*Bai*		12	—	—	—
*Dru*			—	—	—
*Sat*			5	120 (240)	75.00
*Bai*	FIR	6	1	12 (48)	75.00
*Dru*			22	264 (528)	66.67
*Sat*			2	24 (96)	83.33
*Bai*		12	4	96 (192)	75.00
*Dru*			—	—	—
*Sat*			9	216 (432)	66.67

^*a*^The number of test trials is given alongside the overall number of trials (test+ control) in parentheses.

^*b*^The best performance can be lower than 75% for individuals who never reached the criterion. These individuals were not tested in the subsequent tasks, as indicated by the (—) symbol.

### 4.2 Familiar individual recognition

Individual performances are shown (table 1). Two individuals reached 75% success in the first phase after an average of 1.5 sessions (*Sat* after two sessions and *Bai* after one session). The third individual (*Dru*) reached 66.67% success on session 14 but failed to improve afterwards. *Bai* and *Sat* were then moved to a second phase with novel stimuli. *Bai* completed the task after four sessions. *Sat* reached 66.67% success in her first session but failed to improve afterwards. After eight sessions, *Sat* refused to take part in the experiment. Again, there were no obvious consistent differences in the number of sessions each individual required to reach their maximum performances (table 1).

### 4.3 Comparison of familiar and unfamiliar individual recognition

**Figure 4.**
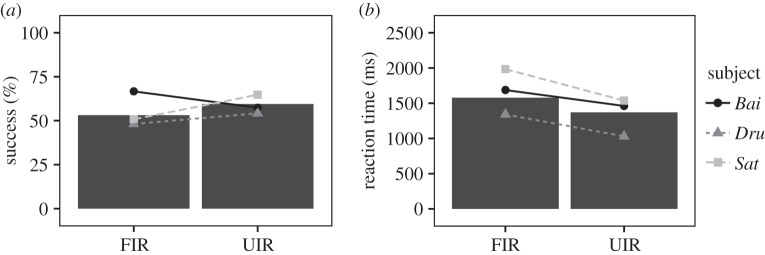
(*a*,*b*) Comparison of subjects' performances in the unfamiliar (UIR) and familiar (FIR) recognition tasks. Bars represent the mean performance across all sessions and points show each individual's mean performance across all sessions. Log of reaction times were back-transformed to time (ms).

The model investigating subjects' responses depending on the type of task, the number of repetitions for each stimulus set and the stimulus set identifier was significantly different from the corresponding null model (LRT full versus null model; accuracy: *χ*^2^=86.01, d.f.=25, *p*<0.001; reaction time: LRT: *χ*^2^=51.46, d.f.=25, *p*=0.001), indicating the good explanatory value of these predictors. The macaques' performances with familiar faces did not differ from their performances with unfamiliar faces (accuracy: *β*=0.039, s.e.=0.503, *p*=0.938; reaction time: *β*=0.014, s.e.=0.169, *p*_MCMC_=0.923, figure 4).

**Figure 5.**
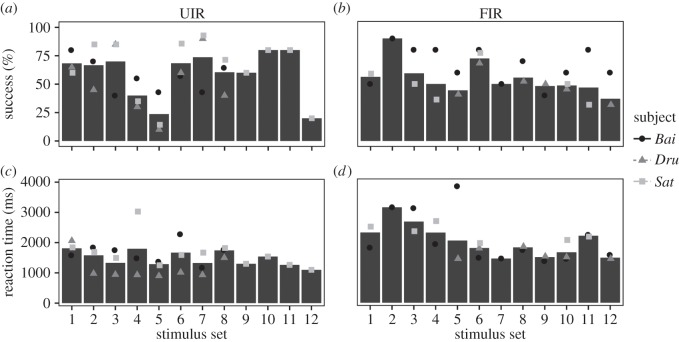
Mean (bars) and individual (dots) performances for each stimulus set in the unfamiliar (UIR (*a*,*c*)) and familiar (FIR (*b*,*d*)) recognition tasks.

Overall, stimulus set was a significant predictor of accuracy (LRT: *χ*^2^=79.70, d.f.=22, *p*<0.001), but not reaction time (LRT: *χ*^2^=20.02, d.f.=22, *p*=0.582). In the UIR task, some stimulus sets were associated with a lower probability of success than others (stimulus sets 4, 5 and 12, figure 5). In the FIR task, some stimulus sets were associated with a higher probability of success than others (stimulus sets 2 and 6, figure 5). Visual inspection of these stimulus sets did not reveal any obvious differences that could account for the low success rate on these trials (electronic supplementary material, figure S1).

**Figure 6.**
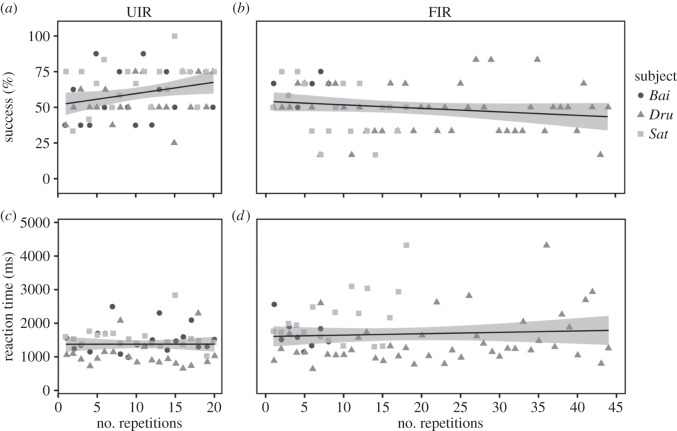
Changes in accuracy (*a*,*b*) and reaction time (*c*,*d*) as a function of the number of repeated trials for the unfamiliar (UIR (*a*,*c*)) and familiar (FIR (*b*,*d*)) recognition tasks. The shaded area around the line represents the 95% CI.

Repeated exposure to the same trials in the UIR task tended to be associated with an increase in subjects' accuracy (*β*=0.036, s.e.=0.021, *p*=0.089, figure 6) but not reaction time (*β*=−0.006, s.e.=0.007, *p*_MCMC_=0.339, figure 6). By contrast, subjects' accuracy decreased after repeated exposure to the same stimulus set in the FIR task (*β*=−0.016, s.e.=0.008, *p*=0.044, figure 6) and their reaction time increased with repeated exposure to the same stimulus set in the FIR task (*β*=0.012, s.e.=0.003, *p*_MCMC_<0.001, figure 6).

### 4.4 Effect of relationship quality

**Figure 7.**
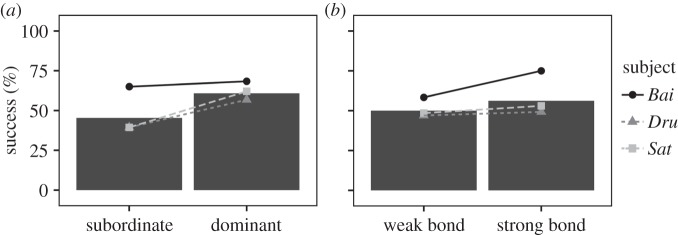
Subjects' performances in the FIR task, according to the quality of the dominance status of the match individual compared with the foil individual (*a*) and according to the strength of the social bond between the subject and the match individual (*b*). Bars represent the mean accuracy and points show each individual's mean accuracy.

We then examined the pattern of response of the three individuals depending on the social status of the individuals depicted on the match and the foil (match individual dominant or subordinate over the foil individual). We also looked at the influence of the quality of the relationship between the subject and the individual depicted as the sample individual (subject having a stronger bond with the match individual or with the foil individual). The set of predictor variables used in our model had a significant influence on the probability of making the correct choice (LRT full versus null model: *χ*^2^=13.35, d.f.=2, *p*=0.001). Indeed, subjects had a bias towards higher ranking familiar individuals (figure 7); they were more successful when the match depicted an individual higher ranking than the individual depicted in the foil (*β*=0.613, s.e.=0.183, *p*<0.001). This effect of dominance status was not statistically significant in the UIR task, although subjects tended to favour lower ranking unfamiliar individuals (higher ranking: mean±s.d.=60.51±48.96%; lower ranking: mean±s.d.=71.43±45.50%; *β*=−0.487, s.e.=0.289, *p*=0.092). In the FIR task, subjects' performances were similar regardless of whether they shared a strong or weak bond with the match individual (*β*=0.143, s.e.=0.183, *p*=0.434, figure 7).

**Figure 8.**
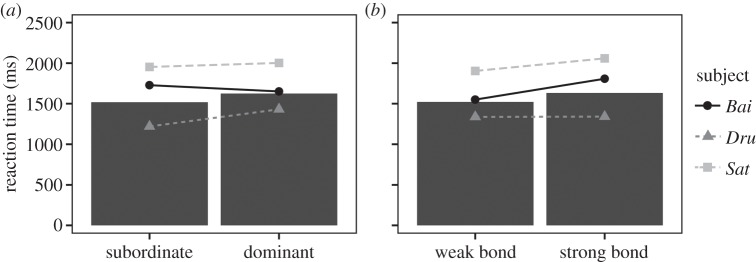
Subjects' reaction times in the FIR task, according to the quality of the dominance status of the match individual compared with the foil individual (*a*) and according to the strength of the social bond between the subject and the match individual (*b*). Bars represent the mean reaction time and points show each individual's mean reaction time. Reaction times were back-transformed to time (ms).

When looking at the effect of the same social variables on the reaction time in the FIR task, the model with predictors was not significantly different from the corresponding null model (LRT full versus null model: *χ*^2^=1.61, d.f.=2, *p*=0.446), indicating the poor explanatory value of these predictors for the reaction time (dominance: *β*=0.063, s.e.=0.073, *p*_MCMC_=0.389; social bond: *β*=0.062, s.e.=0.072, *p*_MCMC_=0.388; figure 8). In the UIR task, the model with dominance status as a predictor of reaction time was significantly different from the corresponding null model (LRT full versus null model: *χ*^2^=34.83, d.f.=1, *p*<0.001). However, the effect of dominance status, as a factor, only approached significance (*β*=0.143, s.e.=0.077, *p*_MCMC_=0.064). Subjects tended to take longer to complete a trial if the sample and match individual was higher ranking than the foil individual (higher ranking: mean±s.d.=1691.36±1229.07 ms; lower ranking: mean±s.d.=1318.61±569.60 ms).

## 5. Discussion

Our results show that crested macaques can discriminate between faces of individuals they have never seen prior to the experiment as well as faces of individuals with whom they interact on a daily basis. Although rhesus macaques were tested using the same paradigm [34,35], our small sample size, our experimental setting (group-housed animals and voluntary participation) and the number of stimuli used were different from Parr's studies which makes direct comparison difficult and potentially inaccurate. Nevertheless, the performances of the crested macaques fall within the range of those reported for rhesus macaques [34,35].

Perhaps our most interesting finding was that all three subjects were sensitive to social characteristics of the familiar individuals represented in the pictures. The dominance status of the sample individual significantly influenced the response of the subjects as indicated by significantly higher percentages of success when the sample and match represented a familiar individual of higher ranking than the foil individual. The macaques also tended to take longer to respond when viewing faces of unfamiliar high-ranking individuals, which could suggest that they can perceive some information regarding the social status of unfamiliar individuals using facial cues only. Although with our small sample size it is inappropriate to draw firm conclusions, these results seem to fit with previously published findings. Dominance status is known to be an important feature of macaques' societies and it can have robust effects in cognitive experiments. For example, rhesus macaques prefer to view faces of high-status but not low-status individuals [29,31]. Social status also modulates gaze following responses in rhesus [30] and long-tailed macaques [40]. This sensitivity to social status is probably the result of selective attention to the faces of those individuals who are the most relevant to the observer. This influence of social status seems logically important for more despotic species such as rhesus and long-tailed macaques. However, it was not expected for more tolerant species, such as crested macaques, where power asymmetries are usually more balanced and dominance relationships less influential [36,52,53]. Instead, and in accordance with previous results, we expected social bonds to be more influential in this species [25,32]. The dominance bias observed in our study was even more surprising given that in a gaze following study involving the same group of crested macaques, dominance status had no effect on gaze following response while the strength of the social bond did [25]. It should be noted though, that these two studies focused on different aspects of social attention. While dominance status did not affect the gaze following responses, individuals might still have monitored high-ranking group mates more often than low-ranking ones [54,55]. This attention bias might not have had an impact on the success and latency of gaze following. Additionally, this inconsistency could be the result of radical demographical changes that occurred within this social group since the gaze following study (i.e. death of two individuals), which could have affected the individuals and the social dynamic within the group [56,57]. It is also possible that this social bias is simply a by-product of our small sample size and that it would disappear if we had tested more individuals.

Another novelty of our study was the ability to directly compare the performances of the same individuals tested with both unfamiliar and personally familiar faces. A traditional prediction made when investigating the effect of familiarity in face processing studies is that subjects should perform better with familiar faces [12,28]. Visual representation of personally familiar faces are thought to be accompanied by rich, semantic, episodic and emotional information about the individual represented on the picture which should facilitate better recognition of that individual [14,15]. Surprisingly, in our study, crested macaques' performances (accuracy and reaction time) were not different when discriminating familiar faces compared with unfamiliar faces. One possible explanation is that the crested macaques did not recognize the familiar individuals but merely treated the pictures as complex visual stimuli. However, this is unlikely as the macaques seemed to discriminate the familiar individuals by dominance status. Moreover, if the macaques treated the pictures as complex visual stimuli, then it is likely that their performances would have increased after repeated exposure to the same stimuli, as a result of learning. Our analysis revealed only a modest improvement in subjects' accuracy after up to 20 repetitions of the same unfamiliar faces and a significant decrease in accuracy after up to 44 repetitions of the same familiar faces. Rather than indicating a learning effect, this pattern suggests that subjects habituated to the stimuli and lacked interest in the task. As the experiment went on, subjects were more difficult to test, preferring to remain with their group mates instead of engaging with the task. We therefore suggest that the crested macaques tested in this study applied their knowledge of the social attributes of the real individuals to their pictorial representations, as suggested for other primate species [18,20–22].

The lack of difference in accuracy and reaction time between familiar and unfamiliar faces could indicate that unfamiliar faces are as relevant to crested macaques as faces of their group mates. In the wild, crested macaques regularly encounter neighbouring groups and male migrations between groups are frequent (A Engelhardt 2006–2015, unpublished data). In such fluid social systems, quickly identifying unfamiliar individuals can be crucial [58–61]. Capuchin monkeys tested in a similar task were even more successful with unfamiliar faces than familiar ones, possibly because of the novelty such stimuli provide [9]. So far, only chimpanzees displayed the same familiar face advantage as humans, possibly because of the fission–fusion nature of chimpanzees' social organization [62]. In Parr and co-workers' study, the subjects were not personally familiar with the individuals; rather, the same stimuli were used as in a number of prior studies and so the chimpanzees knew the faces but had never interacted with the individuals. Such differences make comparison between crested macaques and chimpanzees difficult, but they do suggest that future studies should address variation in subjects' performances according to different degrees of familiarity (i.e. unfamiliar, familiarized and personally familiar). It is also worth mentioning that the differences between primates' performances with unfamiliar and familiar faces are best revealed when stimuli are presented across viewpoints [12]. As we used a mix of congruent and incongruent head orientation in both tasks, our design might not be sensitive enough to reveal differences in the processing of familiar and unfamiliar faces.

The failure to reach criterion for some individuals could be due to the size of the stimulus set we used. A number of studies have shown that capuchins' and rhesus macaques' ability to form and use an identity concept to solve a MTS task increases with the number of stimulus sets used [63,64] as they learn to generalize across stimuli. A possible alternative, lower level explanation is that the macaques tested in our study learnt the visual appearance of the stimulus sets and used associative learning to complete the task. However, if this was the case, then the percentage of success should be a lot higher as the number of associations to remember is not high and macaques possess good learning abilities [65]. By contrast, if the macaques did not understand the task at all and were only responding randomly then their performances would be centred around 50%. Although not extremely high, the performances of the macaques tested in this study were consistently above chance level of 50%. However, the macaques' variation in performance might be due to relative novelty of our experimental setting and the general procedures we used. Specifically, we tested animals who belonged to a social group and whose group mates were often waiting near the testing area (outdoor platform, see figure 1). These social distractions were not avoidable in this study and may impact negatively on the macaques' performances. In addition, for dietary reasons, we were limited in the number of trials we could conduct each day, and in the types of food items that we could use as rewards. All these reasons could have reduced the subjects' motivation and therefore lowered their performances. Although problematic, these compromises might be unavoidable when working with novel animals in zoo settings as an alternative to animals available in laboratories.

A substantial body of work suggests that macaques rely more on the general configuration of the basic features of faces (i.e. first-order configuration) rather than the relative arrangement of the facial features with regards to one another (i.e. second-order configuration), whereas chimpanzees seem to rely on a more holistic processing of faces, similarly to humans [5,7]. Given the close phylogenetic relationship between different macaque species, it is likely that these results would also apply to crested macaques, although future studies are needed to test this hypothesis. In this respect, the use of group-living animals appears to be a rare and valuable resource for cognitive research. In addition to the improvement that group-housed testing implies in terms of animal welfare [66–70] and public engagement with science [71–73], it also dramatically increases the array of species that can be studied and the range of scientific questions that can be addressed. Not only can this type of cognitive testing be implemented in public institutions like zoos with species rarely available in traditional research facilities [74], but it also allows us to easily use stimulus material from the social domain and, thus, tackle important questions in the field of social cognition [75]. Although this method can be time-consuming, sometimes requires specific equipment [66,75,76], and is often criticized for the lack of control it allows over the testing conditions, it can yield results comparable to individual laboratory-based testing [69,75]. This consistency is confirmed by the similar results obtained by the socially housed crested macaques tested in this study and pair-housed rhesus macaques tested with the same paradigm [34].

## Supplementary Material

ESM1. Training procedures and performances

## Supplementary Material

ESM2. Stimuli used in the familiar and unfamiliar individual recognition tasks.

## Supplementary Material

ESM3. Individual performances.

## Data accessibility

All data used in the analyses has been uploaded online as electronic supplementary material.
