# Supplementary material for: Familiar and unfamiliar face recognition in crested macaques (Macaca nigra)
Source: R Soc Open Sci. 2015 May 27;2(5):150109. doi: 10.1098/rsos.150109 (PMC4453246; doi:10.1098/rsos.150109)
Supplement: ESM1. Training procedures and performances [file rsos150109supp1.pdf]

# Electronic Supplementary Material
# Familiar and unfamiliar face recognition in crested macaques (*Macaca nigra*)

Jérôme Micheletta, Jamie Whitehouse, Lisa A. Parr, Paul Marshman, Antje Engelhardt, Bridget M. Waller

## I. Training procedures:

1. **Individuals were rewarded for siting outside the testing unit.**
2. **Individuals were rewarded for being in the testing unit.**
3. **Individuals were rewarded for taking food from the experimenter's hand.**
4. **Individuals were rewarded for touching a target held by the experimenter, in front of the food item:**

We used a white plastic cylinder with red ends as target. The item could be held it firmly. The experimenter placed a food item in the palm of the hand, as he would normally to just to give a food reward to the macaque (step 3). Then the hand is closed while holding the target. The target is presented so that the macaques reach for the food item but could not obtain it. He would then try to grab the target to pull the hand closer. When he did, the hand was opened and the food offered to the animal. This was repeated until the macaque modified the initial grabbing action for a more gentle touch. This was indicated by a gesture clearly designed to touch the target instead of trying to reach the experimenter's hand to open it and get the reward.

5. **Individuals were rewarded for touching a target while no food item is held by the experimenter:**

This step was similar to the previous one but the experimenter did not hold a piece of food. The macaques needed to get used to achieve a task when there was no obvious physical link with the food. The rewards were kept out of reach from the animal. The experimenter had to reach for them when the macaque touched the target.

6. **Individuals were rewarded for touching a target while no food item is held by the experimenter:**

This step paved the way for the use of the touch screen. The subject needed to aim for the target when it was presented out of reach, through clear Perspex (the monkey could not touch the target, just aim for its location). This was repeated many times while changing the position of the target every trial.

7. **Individuals were rewarded for touching a picture of the target displayed on the touchscreen:**

We used a picture of the same target used in the previous training stage. The picture appeared at a random position on the screen. Once the animal touched it, the image disappeared and the animal received a food reward. Once they mastered this simple task, we required the macaques to touch the target three times within 5 s to obtain a reward. They all did this spontaneously since they were used to get a food item after pressing the screen once. When no reward was obtained, they just kept touching the screen.

**8. MTS training 1:**

Once the macaques were proficient using the touchscreen, we introduced the MTS procedures in stages. We used a custom program designed in Microsoft Visual Studio 2010. The program recorded the identity of the macaque (manual input), the filename of the stimuli used, the latency to touch the sample, the latency to complete the trial, the number of touches outside the boundaries of the sample, the number of touches outside the boundaries of the match and whether the response was correct or not.

We first displayed a black & white clipart as the sample. When the macaque touched it 3 times the same clipart appeared as the match (Fig. S1). No foil was displayed at this stage. 60 clipart were taken from the Microsoft Office 2003 built-in clipart library. Once the macaque touched the match, a food reward was given and the next trial started after an inter-trial interval of 2 s. There was no wrong answer in this task. The task was repeated until the time to complete the trial was consistently under 5 s and the number of touches outside the target images (sample and match) was consistently under 5. This was determined by visually inspecting plots of the number of touches and latency as a function of trial number in excel.

(Fig. S1)

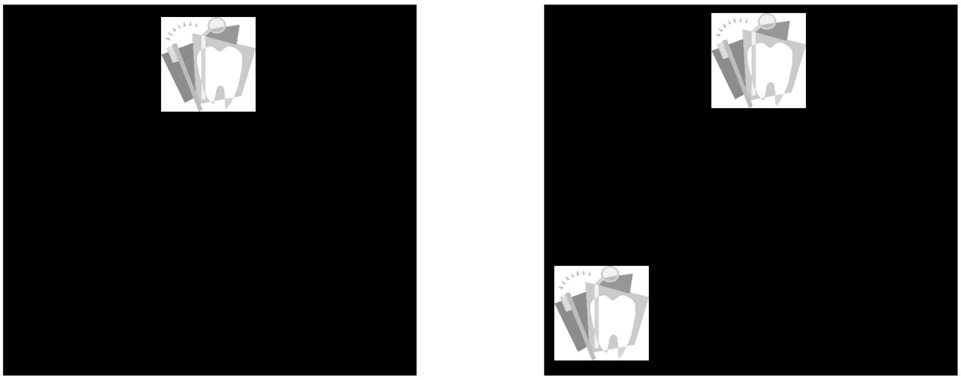

**9. MTS training 2:**

At this stage, we introduced a foil. The foil was a different clipart. At the beginning, we adjusted the size and transparency of the foil to make the task easier: the foil was 50 pixels (instead of 300 for the sample and the match) and 75% transparent (Fig. S2). When the macaques reached 80% of success on 2 consecutive sessions (we had 24 trials sessions) we increased the size of the foil to 100px, 50% transparency then 200px, 25% transparency and finally 300px, 0% transparency.

(Fig. S2)

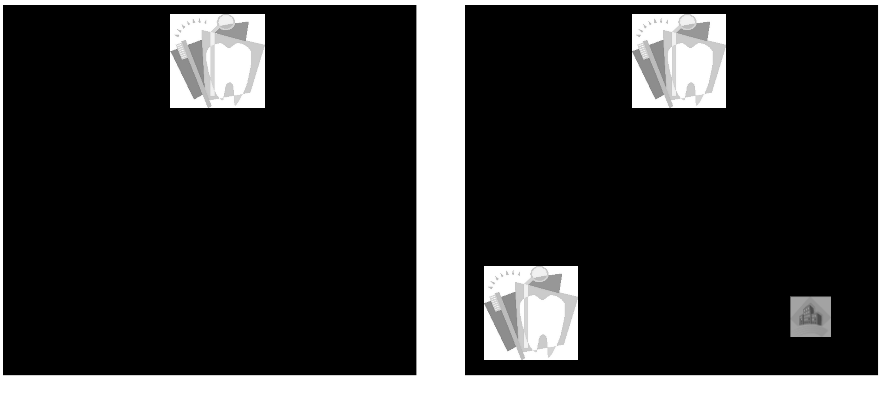

**10. MTS training 3:**

In this stage, the match and the sample were the same picture of an unfamiliar crested macaque while the foil was a clipart (Fig. S3). When the macaque reached 80% on 2 consecutive sessions (24 trials sessions), they were moved to the next stage.

(Fig. S3)

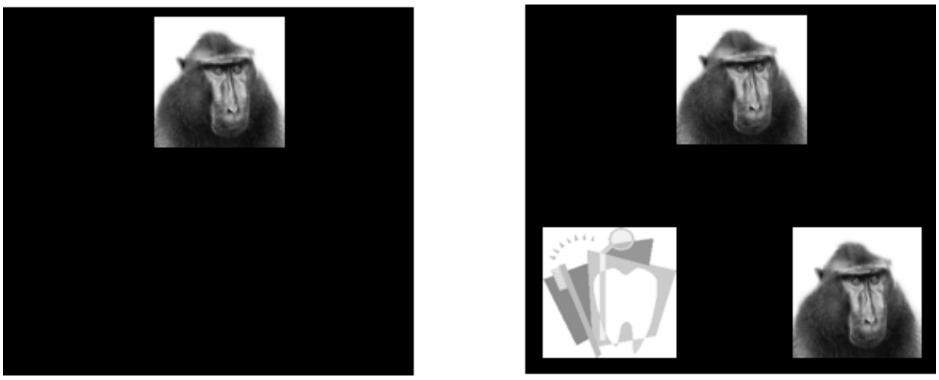

**11. MTS training 4:**

In this stage, the match and the sample were the same picture of an unfamiliar crested macaque while the foil was a clipart (Fig. S4). When the macaque reached 80% on 2 consecutive sessions (24 trials sessions), training was over and they were moved to test phase.

(Fig. S4)

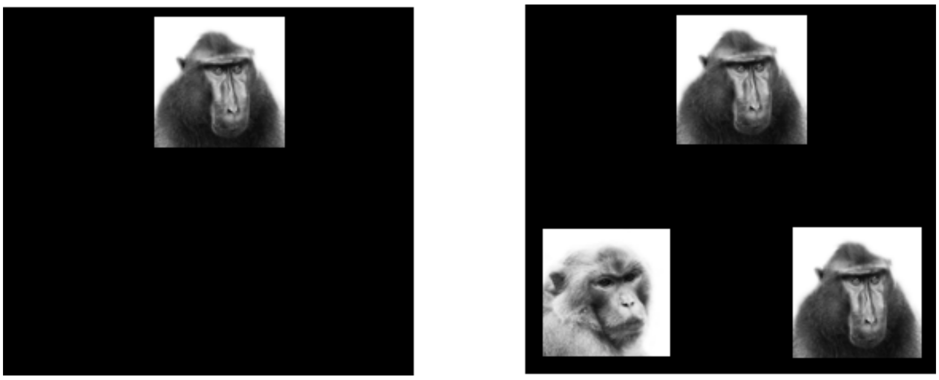

## II. Training performances:

| ID | Task | Number of stimulus sets | Number of sessions to reach criterion (> 80% on 2 consecutive sessions) |
|---|---|---|---|
| Bai | Training 2 (cliparts vs. cliparts) | 24 | 45 |
| Dru | | 24 | 57 |
| Sat | | 24 | 15 |
| Bai | Training 3 (crested macaque vs. cliparts) | 24 | 11 |
| Dru | | 24 | 8 |
| Sat | | 24 | 5 |
| Bai | Training 4 (crested vs. rhesus macaques) | 24 | 4 |
| Dru | | 24 | 10 |
| Sat | | 24 | 4 |
