# Supplementary material for: Familiar and unfamiliar face recognition in crested macaques (Macaca nigra)
Source: R Soc Open Sci. 2015 May 27;2(5):150109. doi: 10.1098/rsos.150109 (PMC4453246; doi:10.1098/rsos.150109)
Supplement: ESM2. Stimuli used in the familiar and unfamiliar individual recognition tasks. [file rsos150109supp2.pdf]

Electronic Supplementary Material - Familiar and unfamiliar face recognition in crested macaques (*Macaca nigra*)

Jérôme Micheletta, Jamie Whitehouse, Lisa A. Parr, Paul Marshman, Antje Engelhardt, Bridget M. Waller

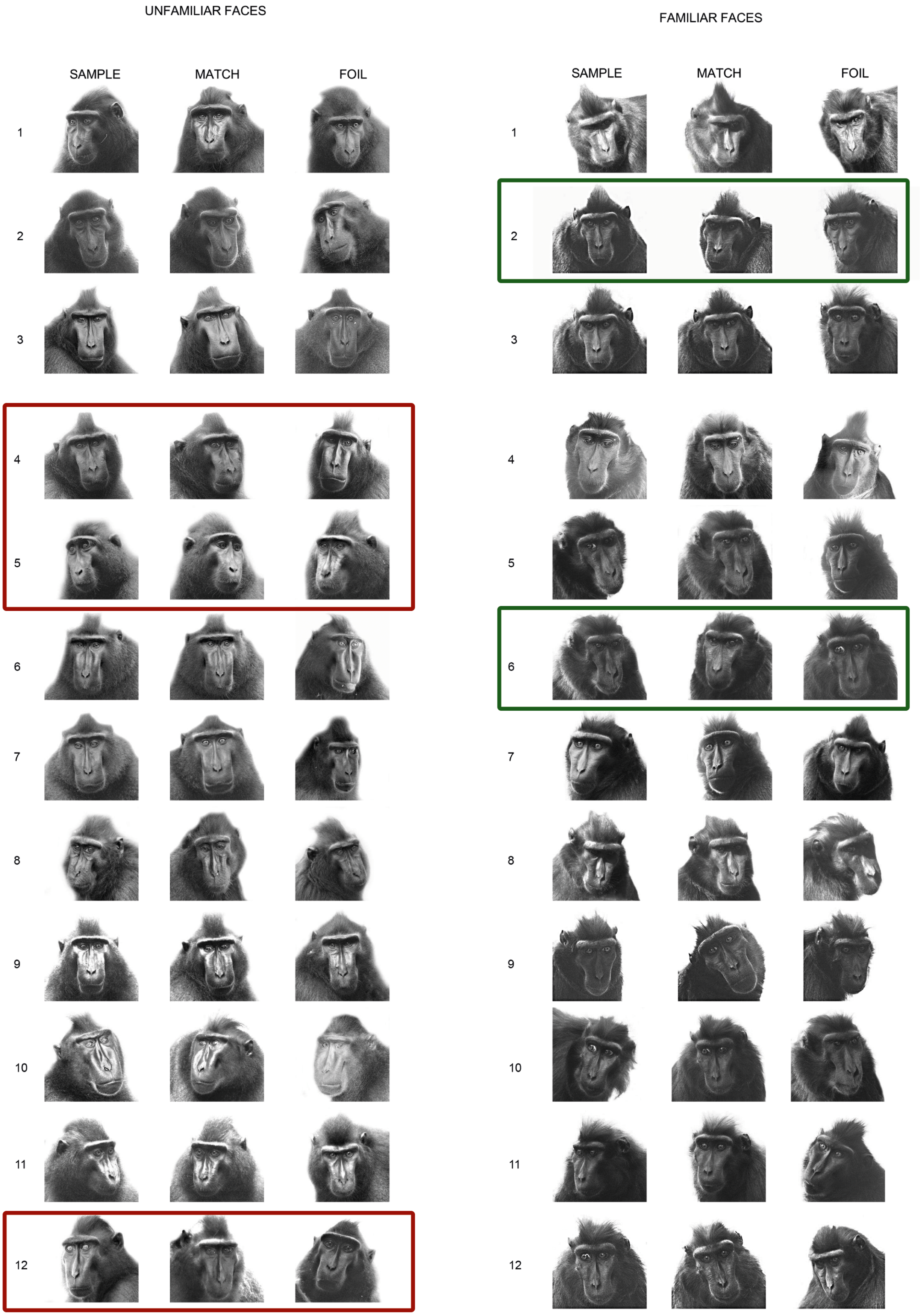

Figure S1. Stimulus sets used in the two MTS experiments. The macaques performed poorly on the stimulus sets framed in red. They were highly successful on the stimulus sets framed in green. See also figure 5 in the paper.
